# DeepFold: enhancing protein structure prediction through optimized loss functions, improved template features, and re-optimized energy function

**DOI:** 10.1093/bioinformatics/btad712

**Published:** 2023-11-23

**Authors:** Jae-Won Lee, Jong-Hyun Won, Seonggwang Jeon, Yujin Choo, Yubin Yeon, Jin-Seon Oh, Minsoo Kim, SeonHwa Kim, InSuk Joung, Cheongjae Jang, Sung Jong Lee, Tae Hyun Kim, Kyong Hwan Jin, Giltae Song, Eun-Sol Kim, Jejoong Yoo, Eunok Paek, Yung-Kyun Noh, Keehyoung Joo

**Affiliations:** Department of Computer Science, Hanyang University, Seoul 04763, Korea; Center for Advanced Computation, Korea Institute for Advanced Study, Seoul 02455, Korea; Department of Computer Science, Hanyang University, Seoul 04763, Korea; Center for Advanced Computation, Korea Institute for Advanced Study, Seoul 02455, Korea; Department of Computer Science, Hanyang University, Seoul 04763, Korea; Center for Advanced Computation, Korea Institute for Advanced Study, Seoul 02455, Korea; Center for Advanced Computation, Korea Institute for Advanced Study, Seoul 02455, Korea; Department of Artificial intelligence, Hanyang University, Seoul 04763, Korea; Department of Computer Science, Hanyang University, Seoul 04763, Korea; Center for Advanced Computation, Korea Institute for Advanced Study, Seoul 02455, Korea; Center for Advanced Computation, Korea Institute for Advanced Study, Seoul 02455, Korea; Department of Artificial intelligence, Hanyang University, Seoul 04763, Korea; Department of Physics, Sungkyunkwan University, Suwon 16419, Korea; School of Electrical Engineering, Korea University, Seoul 02841, Korea; Standigm Inc., Seoul 06234, Korea; Artificial Intelligence Institute, Hanyang University, Seoul 04763, Korea; Basic Science Research Institute, Changwon National University, Changwon 51140, Korea; Department of Computer Science, Hanyang University, Seoul 04763, Korea; School of Electrical Engineering, Korea University, Seoul 02841, Korea; School of Computer Science and Engineering, Pusan National University, Busan 46241, Korea; Department of Computer Science, Hanyang University, Seoul 04763, Korea; Department of Physics, Sungkyunkwan University, Suwon 16419, Korea; Department of Computer Science, Hanyang University, Seoul 04763, Korea; Department of Computer Science, Hanyang University, Seoul 04763, Korea; School of Computational Sciences, Korea Institute for Advanced Study, Seoul 02455, Korea; Center for Advanced Computation, Korea Institute for Advanced Study, Seoul 02455, Korea

## Abstract

**Motivation:**

Predicting protein structures with high accuracy is a critical challenge for the broad community of life sciences and industry. Despite progress made by deep neural networks like AlphaFold2, there is a need for further improvements in the quality of detailed structures, such as side-chains, along with protein backbone structures.

**Results:**

Building upon the successes of AlphaFold2, the modifications we made include changing the losses of side-chain torsion angles and frame aligned point error, adding loss functions for side chain confidence and secondary structure prediction, and replacing template feature generation with a new alignment method based on conditional random fields. We also performed re-optimization by conformational space annealing using a molecular mechanics energy function which integrates the potential energies obtained from distogram and side-chain prediction. In the CASP15 blind test for single protein and domain modeling (109 domains), DeepFold ranked fourth among 132 groups with improvements in the details of the structure in terms of backbone, side-chain, and Molprobity. In terms of protein backbone accuracy, DeepFold achieved a median GDT-TS score of 88.64 compared with 85.88 of AlphaFold2. For TBM-easy/hard targets, DeepFold ranked at the top based on Z-scores for GDT-TS. This shows its practical value to the structural biology community, which demands highly accurate structures. In addition, a thorough analysis of 55 domains from 39 targets with publicly available structures indicates that DeepFold shows superior side-chain accuracy and Molprobity scores among the top-performing groups.

**Availability and implementation:**

DeepFold tools are open-source software available at https://github.com/newtonjoo/deepfold.

## 1 Introduction

Proteins serve as the essential building blocks of biological organisms, acting as the machinery of life. Protein functionality is highly dependent on their 3D structures; therefore, predicting and understanding these structures is crucial. The prediction of complete protein structures from their amino acid sequences alone has been a long-standing challenge, as demonstrated by the biennial CASP competition since 1994.

Two primary computational approaches have been employed to address this challenge: template-based modeling (TBM) and free modeling (FM). TBM constructs a protein structure by using structures of homologous sequences, initially introduced in a study by Browne *et al.* (Browne *et al.* 1969). In TBM methods, finding a highly similar sequence from a given sequence is critical to producing accurate structures (Pearce and Zhang 2021). A conventional method for this is to align sequences using a position-specific score matrix (PSSM) (Altschul *et al.* 1997), or sequence profile Hidden Markov Models (HMMs) (Krogh *et al.* 1994) which made huge impact on searches for similar sequences and structures. These template structures serve as structural restraints for target structure modeling through sequence-structure alignment and optimization of empirical molecular mechanics force fields (Šali and Blundell 1993, Case *et al.* 2005, Huang *et al.* 2016). Subsequent refinement, particularly of side-chain structures, is performed using empirical potentials employing molecular dynamics refinement or more efficient global optimization algorithms (Joo *et al.* 2007, Hong *et al.* 2017). Numerous groups have contributed to this field up until CASP12 (Joo *et al.* 2007, Adhikari *et al.* 2017, Hong *et al.* 2017, Ovchinnikov *et al.* 2017, Wang *et al.* 2017b, Zhang *et al.*, 2017).

As an alternative, FM does not require known templates and instead exploits thermo-physical properties of protein structures. Molecular dynamics methods are predominant in this area (Zhang *et al.* 2011, Robustelli *et al.* 2018). Based on the assumption that native structures exhibit the lowest energy conformations (Anfinsen 1973), researchers attempt to identify the lowest energy conformations. However, this approach is practical only for short sequence lengths (Duan and Kollman 1998).

Another route to protein structure modeling without template structures has been prediction of contacts between residues which typically utilizes the correlated mutations of residues from multiple sequence alignments for the target sequence (Morcos *et al.* 2011, Ekeberg *et al.* 2013). An important breakthrough has been an idea to incorporate deep neural networks to harness the contact information hidden in the MSA’s (Wang *et al.*2017a, b) which led to the surprising appearance and success of AlphaFold1 in CASP13 (Senior *et al.* 2020). Recently, deep neural network models called AlphaFold2 (Jumper *et al.* 2021) (AF2) and RoseTTAFold (Baek *et al.* 2021) have made significant breakthroughs in this problem. These neural networks use attention modules (Bahdanau *et al.* 2014, Vaswani *et al.* 2017) to inject long-range contact information from MSAs and templates into vector representations, successfully constructing a complete protein structure based on the representation. In CASP14, AF2 demonstrated striking prediction performance, showing high accuracy in predicted protein structures on most of the targets (Kwon *et al.* 2021, Pereira *et al.* 2021). However, despite AF2’s remarkable results on average, there still remain some targets for which the predictions of AF2 are rather poor, especially in the cases when MSAs are insufficient for proper modeling. Therefore there are still rooms for further improvement in deep neural network models, both in terms of efficiency and quality of the predicted structures. In particular, improving the accuracy of side-chain modeling would be important for high accuracy modeling and real applications.

Several attempts have been made to improve AF2 in various ways since its advent. One avenue is to address the efficiency of training and inference. UniFold (Li *et al.* 2022) and OpenFold (Ahdritz *et al.* 2022) are the two initial works in this field. While UniFold is a slightly modified model of AF2 with its own training system, OpenFold is a reconstructed version of AF2 with accelerated training/inference time. ColabFold (Mirdita *et al.* 2022), ParaFold (Zhong *et al.* 2021), and FastFold (Cheng *et al.* 2022) are another model that demonstrated fast MSA searches and inference speed. Some works have attempted to build models that cover certain limitations of AF2. ESMFold (Lin *et al.* 2023), EMBER2 (Weissenow *et al.* 2022), and HelixFold (Fang *et al.* 2023) presented end-to-end models that can predict complete structures without MSAs or templates, which is not possible with AF2. With the recent success of diffusion models (Ho *et al.* 2020, Song *et al.* 2021), various efforts have been made toward predicting protein structures using generative models (Liu *et al.* 2022, Trippe *et al.* 2023, Watson *et al.* 2023). These models showed some potentials but still have limitations compared to AF2-based methods in terms of accuracy and effectiveness.

Even with such a long history and remarkable advances in protein structure prediction, improvement in the quality of detailed structures such as side-chains has always been in strong demand, along with the quality of protein backbone structures, especially in the larger community of life sciences and industry. In this study, we address this problem by constructing a protein structure prediction model called DeepFold. In order to achieve more accurate backbone and side-chains with enhancement of the overall quality of protein structures, we modified the losses of the side-chain torsion angles and FAPE (frame aligned point error). And we introduced new loss functions for side chain confidence and secondary structure prediction. In addition, we replaced the template feature generation of AF2 by integrating a refined version of the alignment method, CRFalign (Lee *et al.* 2022), which builds upon the established principles of conditional random fields (Peng and Xu 2009). This refined version incorporates new feature vectors and gradient boosted regression trees. Lastly, re-optimization of the predicted structures is performed using a powerful global optimization algorithm called conformational space annealing (CSA) (Lee *et al.* 1997, Joo *et al.* 2007). We used a molecular mechanics energy function that integrates the potential energies obtained from the distogram and side-chain prediction. For validation, we benchmarked the prediction protocol of DeepFold on CASP13/14 targets which showed consistent improvement in side-chain torsions of proteins with a modest increase in backbone accuracy. In CASP15 blind competition, DeepFold achieved quite promising result, ranking fourth out of 132 teams in the assessors’ formula metric. Further analysis revealed that DeepFold outperformed top-performing groups in terms of detailed protein structure, including side-chains and Molprobity scores.

## 2 Materials and methods

**Figure 1.**
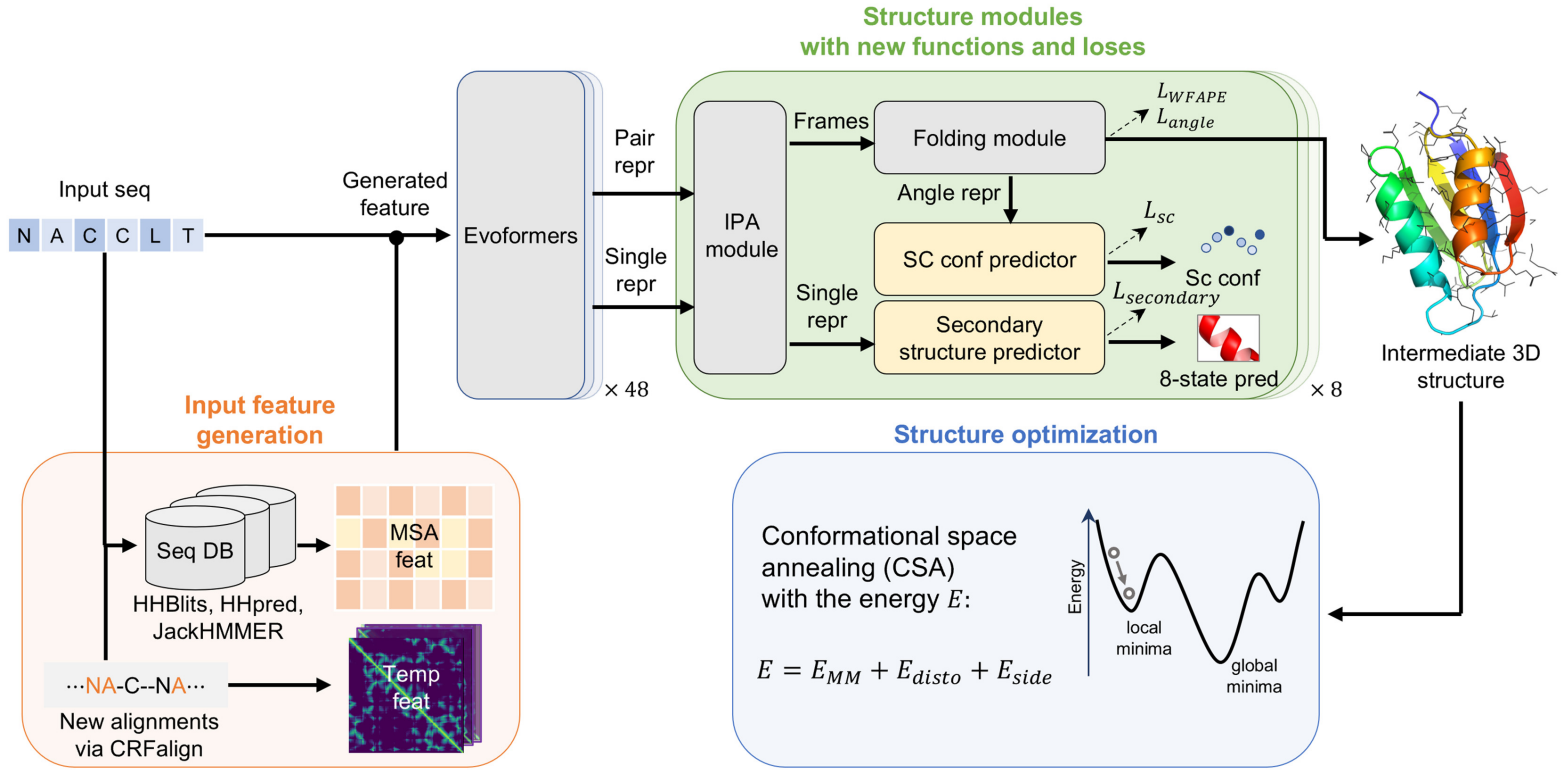
Prediction flow diagram of DeepFold. Given an amino acid sequence, the method first generates input features using MSAs and templates, where the MSAs are obtained from HHBlits, JackHMMER, and HHpred, and the templates/alignments are generated by CRFalign. Protein 3D structures are predicted by DeepFold network and then final structures are re-optimized by conformational space annealing (CSA). (See the main text for details.)

Figure 1 shows the flow diagram of DeepFold for protein structure prediction. Given an amino acid sequence, DeepFold starts with generating MSAs and templates, which are processed as input to the DeepFold network. Then, Evoformer (Jumper *et al.* 2021) network generates single and pair representations for the sequence that are fed into Structure modules to produce protein 3D structures. Those structures are re-optimized by CSA with an energy function including molecular mechanics force field and additional potentials from distogram and side-chain predictions. Details of DeepFold, with focus on modifications of AF2 are described below.

### 2.1 MSA and template feature generation

The input features for DeepFold are generated from MSAs and templates. For the MSAs, DeepFold uses standard MSA generation procedure of AF2 using JackHMMER (Johnson *et al.* 2010) with MGnify (Richardson *et al.* 2023), JackHMMER with UniRef90 (Suzek *et al.* 2015), and HHblits (Remmert *et al.* 2012) with Uniclust30 (Mirdita *et al.* 2017)/BFD (Steinegger *et al.* 2019). As for the templates, we collected template candidates from HHPRED. In addition, we ranked all the structures in the PDB40 database (see Dataset section) based on their structural similarity to our query protein using DeepAlign (Nolle *et al.* 2020) with the AF2 model. From these ranked results, we selected the top 20 structures as additional template candidates. These aggregated template candidates were then re-ranked using CRFalign (Lee *et al.* 2022), a sequence-structure alignment method based on pairwise conditional random fields and gradient boosted regression trees. We utilized up to four of these templates and their respective alignments as input for the DeepFold network. In protein structure prediction, templates have been shown to be especially beneficial for targets with similar templates (Wu *et al.* 2022).

### 2.2 Sequentially conditioned torsion angle loss

Since the side-chain torsion angles $\chi^{1},\ldots ,\chi^{4}$ are sequentially dependent in terms of 3D coordinates, we modified the torsion angle loss considering this sequential relationship in side chain angles by introducing the pre-torsion angle error ${\tilde{E}}_{\chi^{k}}$:


(1) $${\tilde{E}}_{\chi^{k}}:=\left({\tilde{E}}_{\chi^{k-1}}+E_{\chi^{k}}-\frac{{\tilde{E}}_{\chi^{k-1}}\cdot E_{\chi^{k}}}{\sqrt{2}}\right),\;\text{for}\;k=2,3,4,$$

with the initial condition ${\tilde{E}}_{\chi_{1}}=E_{\chi^{1}}$, where the $E_{\chi^{k}}$ is the square-root angle error defined as:


(2) $$E_{\chi^{k}}:=\sqrt{||\alpha^{\chi^{k}}-{\hat{\alpha }}^{\chi^{k}}|{|}_{2}}\;\text{for}\;k=1,2,3,4.$$

Here, $\alpha^{\chi^{k}}\in R^{2}$ is a cosine/sine vector representation of $\chi^{k}$ in a single residue:


(3) $$\alpha^{\chi^{k}}={\left(\begin{array}{c} \;\text{cos}\;\chi^{k},\;\text{sin}\;\chi^{k} \end{array}\right)}^{⊤}.$$

The sequential properties of the pre-torsion angle error ${\tilde{E}}_{\chi^{k}}$ in Equation (1) can be seen as follows. In the case of $\chi_{1}$ where no preceding angles exist, the error is reduced to just the angle error $E_{\chi^{1}}=\sqrt{||\alpha^{\chi^{1}}-{\hat{\alpha }}^{\chi^{1}}|{|}_{2}}$. For the case of $\chi^{2}$, $\chi^{3}$, or $\chi^{4}$, the error references the previous error ${\tilde{E}}_{\chi^{k-1}}$ in the calculation of the current error, such that the current error ${\tilde{E}}_{\chi^{k}}$ increases (i.e. is nonzero) even if the current angle difference $E_{\chi^{k}}$ is zero. Therefore, even though the model predicts correctly $\chi^{2}$ angles, if it predicts $\chi^{1}$ incorrectly, the torsion error of $\chi^{2}$ will be increased. Lastly, summing the pre-torsion angle error ${\tilde{E}}_{\chi^{k}}$ for each of the χ angles, we can define the overall angle loss as follows


(4) $$L_{\text{angle}}=\frac{1}{N}\left(\Sigma E_{\chi^{1}}+\Sigma {\tilde{E}}_{\chi^{2}}+\Sigma {\tilde{E}}_{\chi^{3}}+\Sigma {\tilde{E}}_{\chi^{4}}\right),$$

where *N* is the number of residues and the summations are over the numbers of *k*th side-chain torsion angles respectively (k=1⋯4).

### 2.3 The weighted FAPE loss

The Frame-aligned point error (FAPE) in AF2 plays an essential role in aligning atom positions in the generated rigid frame of each residue (i.e. the predicted local frame of each residue). The FAPE loss of AF2 is defined as


(5) $$L_{\text{FAPE}}(T_{i},x_{j})=\min\left(||T_{i}^{-1}\cdot x_{j}-T_{i}^{-1(\mathrm{true})}\cdot x_{j}^{\left(\mathrm{true}\right)}|{|}^{2},10Å\right),$$

where $T_{i}=(R_{i},{\vec{t}}_{i})$ is the predicted local frame (which represents the rotation $R_{i}$ and translation ${\vec{t}}_{i}$ of *i*th residue) and atom coordinates $x_{j}\in R^{3}$, which measures the squared error between local coordinates $T_{i}^{-1}\cdot x_{j}$ and its ground truth $T_{i}^{-1(\mathrm{true})}\cdot x_{j}^{\left(\mathrm{true}\right)}$. Note that the loss has a clamping value of 10 Å that washes out the loss values larger than 10 Å. In DeepFold, we introduce the weighted FAPE loss as follows:


(6) $$L_{\text{WFAPE}}(T_{i},x_{j})=w_{ij}L_{\text{FAPE}\;}(T_{i},x_{j}),$$

where the weight $w_{ij}$ is determined by following sigmoid-type function


(7) $$w_{ij}=\frac{1}{\left(1+\text{exp}(-2(-d_{ij}+v))\right)}+h,$$

in which $d_{ij}$ is the distance with maximal probability from the distogram, between $C_{\beta }$ atoms of residues *i* and *j*. The hyperparameters *v*, *h* control the inflection point of the sigmoid function and an additional weighting constant, respectively. In this work, we used v=12.0, and h=1.5 which were determined by iterative grid searches. This modified loss gives larger weight on the closer atoms.

### 2.4 Secondary structure loss

We introduce secondary structure loss in DeepFold network by implementing small multi-layer perceptrons in the structure module as shown in Fig. 1. This network uses single representations as inputs which were obtained by IPA (Invariant Point Attention) in structure module (Jumper *et al.* 2021). And they return as output the prediction results of 8-state secondary structure of each residue (Kabsch and Sander 1983). To train this network, we defined a loss function for the secondary structure by cross-entropy as follows:


(8) $$L_{\text{secondary}}=-\frac{1}{N}\sum_{i}^{N}\sum_{c=1}^{8}y_{i}^{c}\;\text{log}\;p_{i}^{c},$$

where $y_{i}^{c}$ is the true secondary structure of *i-*th residue with *c*-state, and $p_{i}^{c}$ is the predicted probability that the 8-state secondary structure of the *i-*th residue is in *c*-state.

### 2.5 Side-chain confidence loss

Similarly to the plddt in AF2, we defined another confidence measure focussing on the side chains of protein structure as follows. Side chain confidence $s_{i}$ for each residue i=1,…,N is defined as:


(9) $$s_{i}=\frac{1}{1+{\left(\delta_{i}/\delta_{0}\right)}^{2}}$$

where $\delta_{i}$ is the difference between the true and predicted $\chi^{1}$ angles $\delta_{i}=\chi_{i}^{1(\mathrm{true})}-\chi_{i}^{1(\mathrm{pred})}$. Here, $\delta_{0}$ is a reference value of angle difference which was set as $12^{{}^{\circ}}$ in this work. In order to predict $s_{i}$, we implemented another multi-layer perceptron network in the structure module which returns as output the probability ${\hat{p}}_{i}^{s}$ for $s_{i}$ to be in the specific bin among 50 bins in the interval [0,1]. This can be compared with the true values of $s_{i}$, from which a loss function can be formed. The loss function is defined as the cross-entropy loss between one-hot encoded $s_{i}$ and predictions:


(10) $$L_{\text{sc}}:=-\frac{1}{N}\sum_{i=1}^{N}p_{i}^{s}{}^{⊤}\;\text{log}\;{\hat{p}}_{i}^{s},$$

where the $p_{i}^{s}$ and ${\hat{p}}_{i}^{s}$ are one-hot encoded representations of $s_{i}$ and prediction ${\hat{s}}_{i}$, respectively. In this work, the side chain confidence score for a single sequence is defined by the average score over all residues.

With the modified loss functions and newly added loss terms, we arrive at the following combined loss L which can be used for training the DeepFold model:


(11) $$L=c_{1}L_{\text{angle}}+c_{2}L_{\text{WFAPE}}+c_{3}L_{\text{secondary}}+c_{4}L_{\text{sc}}+c^{⊤}L_{\text{others}},$$

where the $L_{\text{others}}=(L_{\text{aux}},L_{\text{dist}},L_{\text{msa}},L_{\text{conf}},L_{\;\text{exp}\;\;\text{resolved}},L_{\text{vio}})$ are the other losses in AF2 with the default weights c=(0.5,0.3,2.0,0.01,0.01,1.0) as they used in (Jumper *et al.* 2021). Supplementary Table S1 shows the relative weights of various loss terms in our modifications.

### 2.6 Dataset, training, and validation

We used the latest PDB database (Feb. 2022) for training. We clustered the sequences of PDB using CD-HIT with 40% sequence identity, which resulted in 31,911 protein chains. We further filtered 23,366 chains of high-resolution (with < 2.5 Å) for a fine-tuning dataset. The obtained sequences were cropped to 256 and 384 residue sizes as in the AF2 for training. Five DeepFold models were selected from training with various training schedules. For all the trained models, we employed the Uni-fold (a trainable version of AF2) training system, where the trainings were started from the AF2 parameters and then further optimized in the style of transfer learning. Full details are described in Supplementary Section S1.

### 2.7 Re-optimization by conformational space annealing

Once 3D structures are inferred from the DeepFold networks, we perform global optimization using conformational space annealing (CSA) with the full atom force field, distance restraints, and side chain torsion restraints generated by the networks. Full details are described in Supplementary Section S2.

## 3 Results

In order to evaluate the performance of DeepFold, we conducted a blind test in the CASP15 competition. As our Group Names, we used DFolding and DFolding-server, where the latter was a server prediction protocol which was performed without the final CSA re-optimization due to the 3-day deadline. We present our results obtained from the official evaluation data, which was provided by CASP15 assessors of Single Protein and Domain Modeling category [15th Community Wide Experiment on the Critical Assessment of Techniques for Protein Structure Prediction (https://predictioncenter.org/casp15/)]. Traditionally, all targets are divided into domains, which are classified as TBM and FM domains. In the CASP15, total of 109 domains, which were officially assessed, consist of 62 TBM domains (including 47 TBM-easy and 14 TBM-hard), and 47 FM domains (8 FM/TBM and 39 FM). We also present our own assessment of 55 domains (39 targets) including 27 TBM, 27 FM domains, and 1 domain that was not classified, for which native structures were publicly available.

The official metric to compare the performance of all Groups is called *sum* Z-score which is a weighted sum of the Z-scores for each of the domains as in the following CASP15 assessor’s formula (Read and Chavali 2007, Mariani *et al.* 2011, Olechnovič *et al.* 2013, Croll *et al.* 2019, Millán *et al.* 2021, Pereira *et al.* 2021) [GDT-HA (Global Distance Test for High Accuracy), reLLG (Relative eLLG score), ASE (Accuracy Self Estimate), LDDT (Local Distance Difference Test), AA (CAD-score, all atoms), SG (The average value of Sphere Grinder Score), SC${}_{\text{error}}$ (Side Chain Dihedral Error Score), Molprobity (Aggregated Molprobity Score), BB${}_{\text{error}}$ (Backbone Dihedral Error Score), DipDiff (The average difference between the local DipScores)],


(12) $$\begin{array}{c} \mathrm{sum}\;Z-\text{score}=\frac{1}{6}(\text{GDT-HA}+\text{reLLG}+\text{ASE})\\ +\frac{1}{16}(\text{LDDT}+\text{AA}+\text{SG}+{\text{SC}}_{\text{error}})\\ +\frac{1}{12}(\text{Molprb}+{\text{BB}}_{\text{error}}+\text{DipDiff}). \end{array}$$

**Figure 2.**
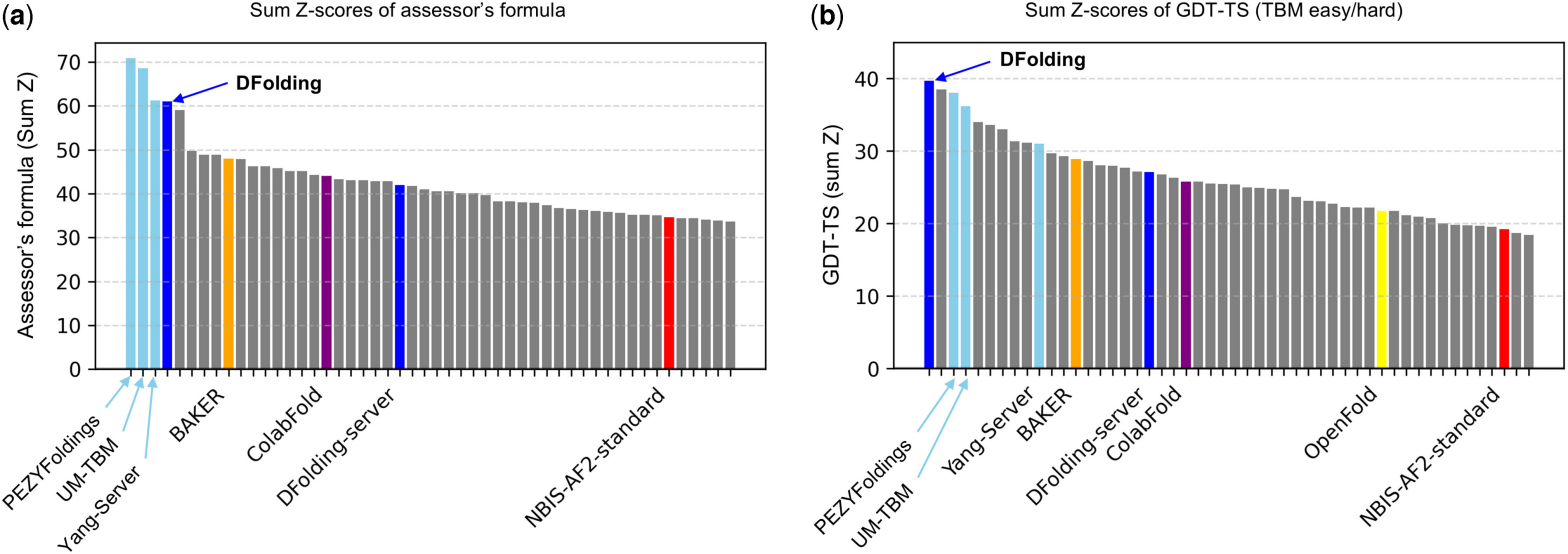
CASP15 rankings in terms of sum Z of (a) the assessor’s formula [Equation (12)] and (b) GDT-TS for TBM easy/hard targets (62 domains in total). In each figure, only top 50 out of 132 teams are shown.

In addition to the above metric, GDT-TS is typically used as a standard measure of modeling accuracy (Global Distance Test for Tertiary Structure) to evaluate the accuracy of global backbone trace of the structure. Figure 2 illustrates the official rankings of DFolding, together with other top 50 among 132 Groups including BAKER (RosettaFold), ColabFold, NBIS-AF2-standard and OpenFold (the latter three are basically standard AF2-based protocols with modification of MSA generation). In Fig. 2a, DFolding was ranked fourth according to the assessor’s formula, while in (b), DFolding was at the top in terms of *sum* Z-score of GDT-TS for 64 domains, classified in the TBM-easy and TBM-hard target. In the figure, DFolding-server was also included, indicating the performance difference in comparison with DFolding. Note that CSA re-optimization is not used in DFolding-server protocol due to the time limit of three-days.

Table 1 shows the results of top 10 performing groups from the Fig. 2a, in addition to three AF2 based methods. All metrics used in assessor’s formula are shown in the average values instead of Z-scores. Despite the differences of backbone accuracies, the values of the metrics such as SC${}_{\text{error}}$, AA, BB${}_{\text{error}}$, and DipDiff are showing very little differences between groups. In particular, the value of SC${}_{\text{error}}$ (which means side-chain error for protein structure models, where lower is better) is not very distinguishable in spite of a significant difference in backbone accuracy such as GDT-TS. This is because the criterion of correctness in the metric of SC${}_{\text{error}}$ is too broad. Below, we will discuss our own evaluation for side-chain accuracies for 55 domains, where native structures are available in public. In the Molprobity score showing protein-likeness, DeepFold achieved a score of 0.97 (lower is better) and performed well among the top groups.

**Table 1.** The official CASP15 results of top 10 performing groups, in addition to three AF2 based methods sorted by assessor’s formula in Equation (12).^a^

Rank	Methods	sum Z	GDT-TS	GDT-HA	reLLG	ASE	LDDT	AA	SG	SC${}_{\text{error}}$	Molprobity	BB${}_{\text{error}}$	DipDiff
1	PEZYFoldings	**70.83**	81.89	68.00	12.65	88.39	0.79	0.77	90.10	9.59	1.34	9.38	0.02
2	UM-TBM	68.56	83.75	**69.49**	12.26	89.04	**0.81**	0.78	**92.06**	**9.42**	1.22	9.21	0.01
3	Yang-Server	61.28	**83.92**	69.48	12.45	85.89	0.79	0.78	91.76	9.50	1.36	9.29	0.02
4	DFolding (DeepFold)	61.07	80.33	66.39	10.53	87.22	0.78	0.76	88.79	9.44	0.97	9.21	0.03
5	Yang	59.01	83.07	68.92	**12.99**	86.13	0.79	0.78	91.01	9.51	1.38	9.29	0.02
6	McGuffin	49.73	79.82	66.34	11.28	85.22	0.77	0.76	88.24	9.43	1.59	9.21	0.01
7	MULTICOM	48.83	80.20	66.41	11.99	75.55	0.78	0.77	89.98	9.44	1.38	9.21	0.01
8	MULTICOM_refine	48.83	79.25	65.46	11.37	85.75	0.79	0.77	89.75	9.43	1.16	9.21	0.01
9	BAKER	47.99	79.33	63.81	8.59	85.72	0.77	0.75	89.20	9.46	**0.75**	9.21	0.03
10	MULTICOM_human	47.90	79.99	66.13	11.08	75.81	0.78	0.76	89.68	9.44	1.38	9.21	0.01
17	ColabFold	44.02	75.36	62.33	10.77	89.75	0.76	0.75	86.57	9.43	1.34	9.21	0.01
45	NBIS-AF2-standard	34.63	75.81	61.74	9.59	**89.84**	0.76	0.75	87.21	9.44	1.28	9.21	0.00
53	OpenFold	33.25	73.71	60.34	9.13	87.79	0.74	0.74	84.72	9.53	1.72	9.30	0.00

a All of the 11 metrics in the formula are also shown as the average (over all the target) of the raw scores. References for each of the predictors listed are as follows: PEZYFoldings (Oda 2023), UM-TBM (Peng *et al.* 2023), Yang-Server (Zheng *et al.* 2023), McGuffin (McGuffin *et al.* 2023), MULTICOM (Liu *et al.* 2023), ColabFold (Mirdita *et al.* 2022), and OpenFold (Ahdritz *et al.* 2022). The top scores in each metric are marked in bold.

**Figure 3.**
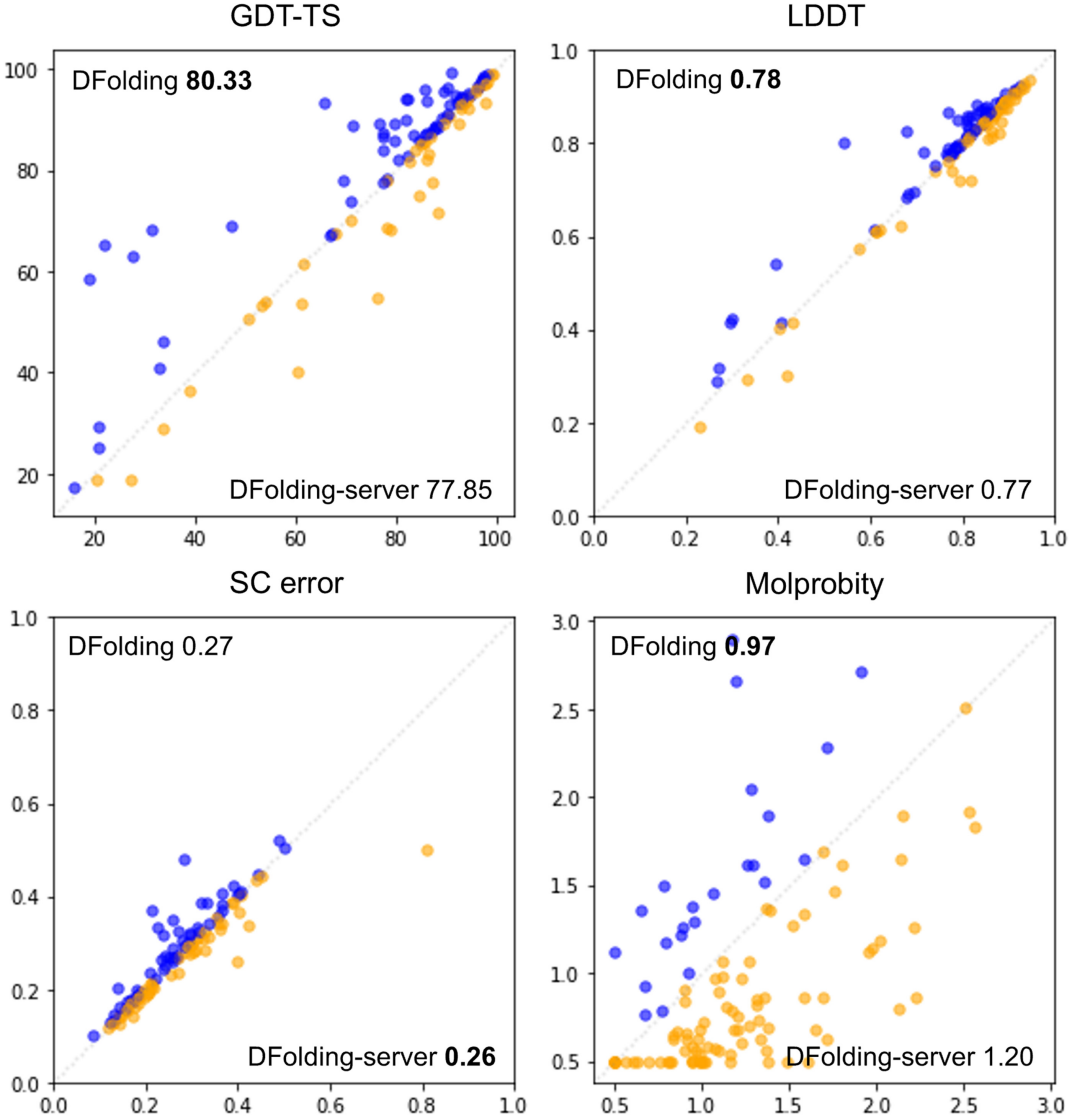
Comparison between the accuracies of DFolding (*y*-axis) versus DFolding-server (without CSA re-optimization, *x*-axis). The CASP15 official results of GDT-TS, LDDT, SC error, and Molprobity are used. Higher values are better for GDT-TS, LDDT, while lower values are better for SC${}_{\text{error}}$ and Molprobity. All numbers within the figures are average values over 109 domains.

Figure 3 shows the effect of CSA re-optimization by comparing DFolding (*y*-axis) versus DFolding-server (*x*-axis) in terms of GDT-TS, LDDT, SC error, and Molprobity, where we used the official metrics provided by CASP15 assessors. DFolding prediction exhibits better performance in average scores for GDT-TS, LDDT, and Molprobity, while difference of SC${}_{\text{error}}$ is very minor. It is due to the same reason mentioned above that the criterion of side-chain error is too broad. In GDT-TS, DFolding performed well for the relatively hard targets (GDT-TS less than about 70), which can be attributed to the result of CSA re-optimization. It also shows significant improvement in Molprobity score.

We now apply further analysis of our own on the performance of DeepFold with the 55 domains for which the native structures are available. In Table 2, GDT-TS and GDT-HA were calculated by using the TM-score program instead of using the official data. Side-chain accuracies of $\chi^{1}$ and $\chi^{2}$ were calculated by using 10° criterion of correctness, instead of using SC${}_{\text{error}}$ used in the official results. All values presented in the table are cumulative Z-scores for each metric, in line with the CASP15 assessor’s methodology (higher values are better). The results from DFolding demonstrate a balanced performance in both backbone and side-chain metrics when compared to leading groups. The table shows that the sum Z-score of DFolding in Molprobity value is significantly higher than all the other top performing groups. Furthermore, the table shows that DFolding achieved higher scores in LDDT, GDT-TS, and GDT-HA than the well-known methods such as AF2, BAKER, ColabFold, and OpenFold.

**Table 2.** CASP15 results of top 10 teams in Table 1, evaluated on 55 domains, for which the native structures are publicly available.^a^

Methods	LDDT	GDT-TS	GDT-HA	$\chi_{\mathrm{acc}}^{1}$	$\chi_{\mathrm{acc}}^{2}$	Molprobity	counts
PEZYFoldings	38.19	51.80	54.93	27.73	27.22	26.99	53
UM-TBM	36.41	47.91	53.86	27.49	31.27	27.71	55
Yang-Server	24.69	49.19	52.44	35.14	35.80	17.34	55
**DFolding (DeepFold)**	31.98	46.76	48.99	29.00	29.42	50.51	55
Yang	22.78	45.03	47.66	33.65	40.38	24.81	55
McGuffin	29.75	44.32	49.21	24.56	29.05	17.03	55
MULTICOM	29.41	38.33	43.88	22.78	27.23	24.20	55
MULTICOM_refine	29.70	28.98	30.73	23.98	23.32	31.21	55
BAKER	15.93	30.73	27.01	35.14	32.44	28.81	55
MULTICOM_human	27.20	37.32	41.77	22.03	22.98	24.34	55
ColabFold	24.93	20.42	21.60	21.40	22.57	21.17	55
NBIS-AF2-standard	21.52	14.87	14.57	22.69	20.29	23.74	54
OpenFold	16.46	14.09	15.16	18.03	21.07	19.67	55

a All scores in the table are Z-sum scores. GDT-TS and GDT-HA in the table are calculated by TM-score (Zhang and Skolnick 2005). The top scores in each metric are marked in bold.

**Figure 4.**
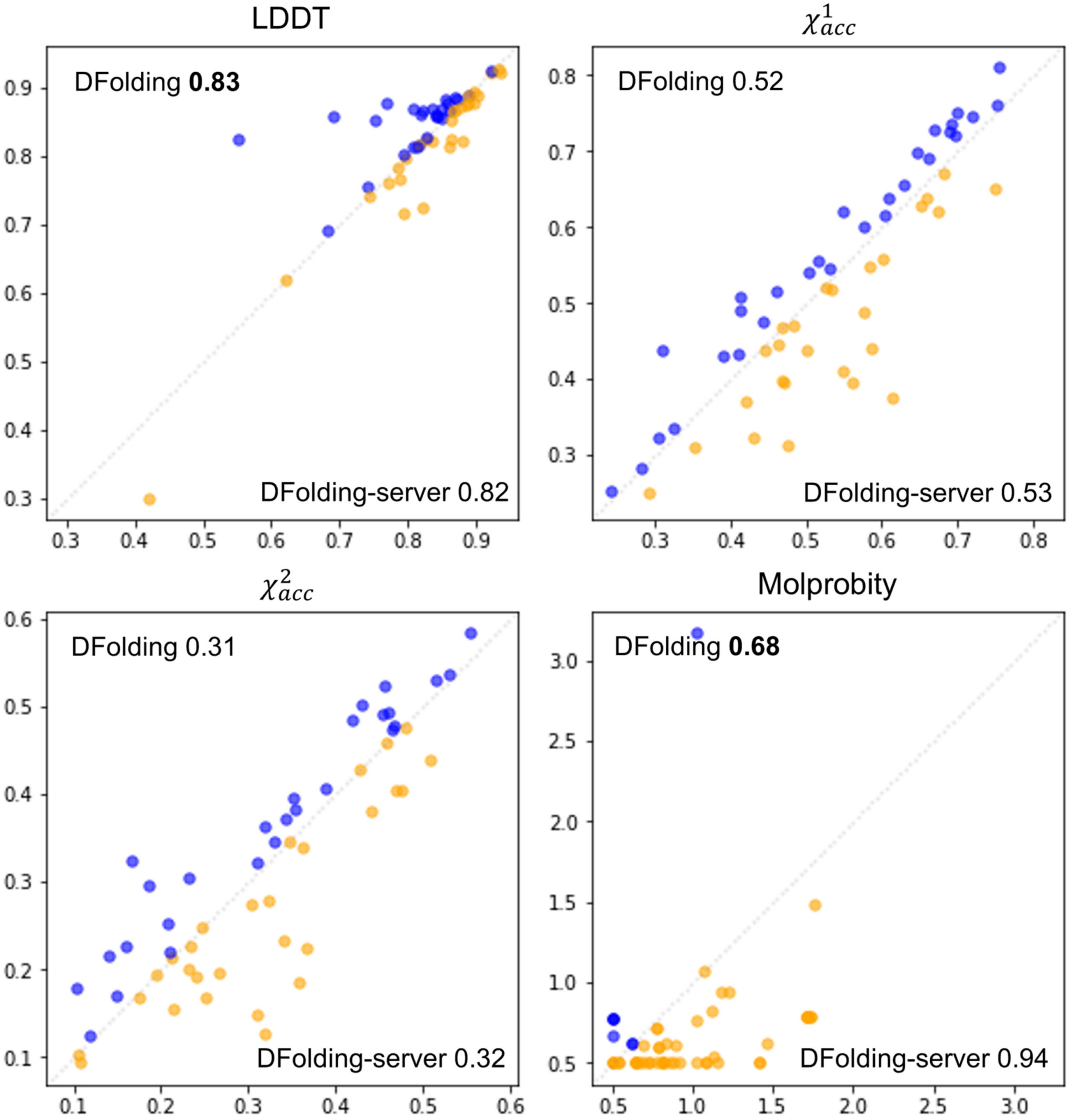
Comparison between the accuracies of DFolding (*y*-axis) and DFolding-server (*x*-axis) for publicly available 55 domains. All numbers within the figures are average values over 55 domains. Lower value is better for Molprobity.

Figure 4 shows a comparison between the accuracies of DFolding and DFolding-server for 55 domains. On average, DFolding outperforms DFolding-server in terms of GDT-TS and Molprobity value, while the sidechain scores are similar in terms of $\chi_{\mathrm{acc}}^{1}$ and $\chi_{\mathrm{acc}}^{2}$. This indicates that CSA re-optimization process of DFolding resulted in a minor improvement for the overall structure of the 55 protein domains.

**Figure 5.**
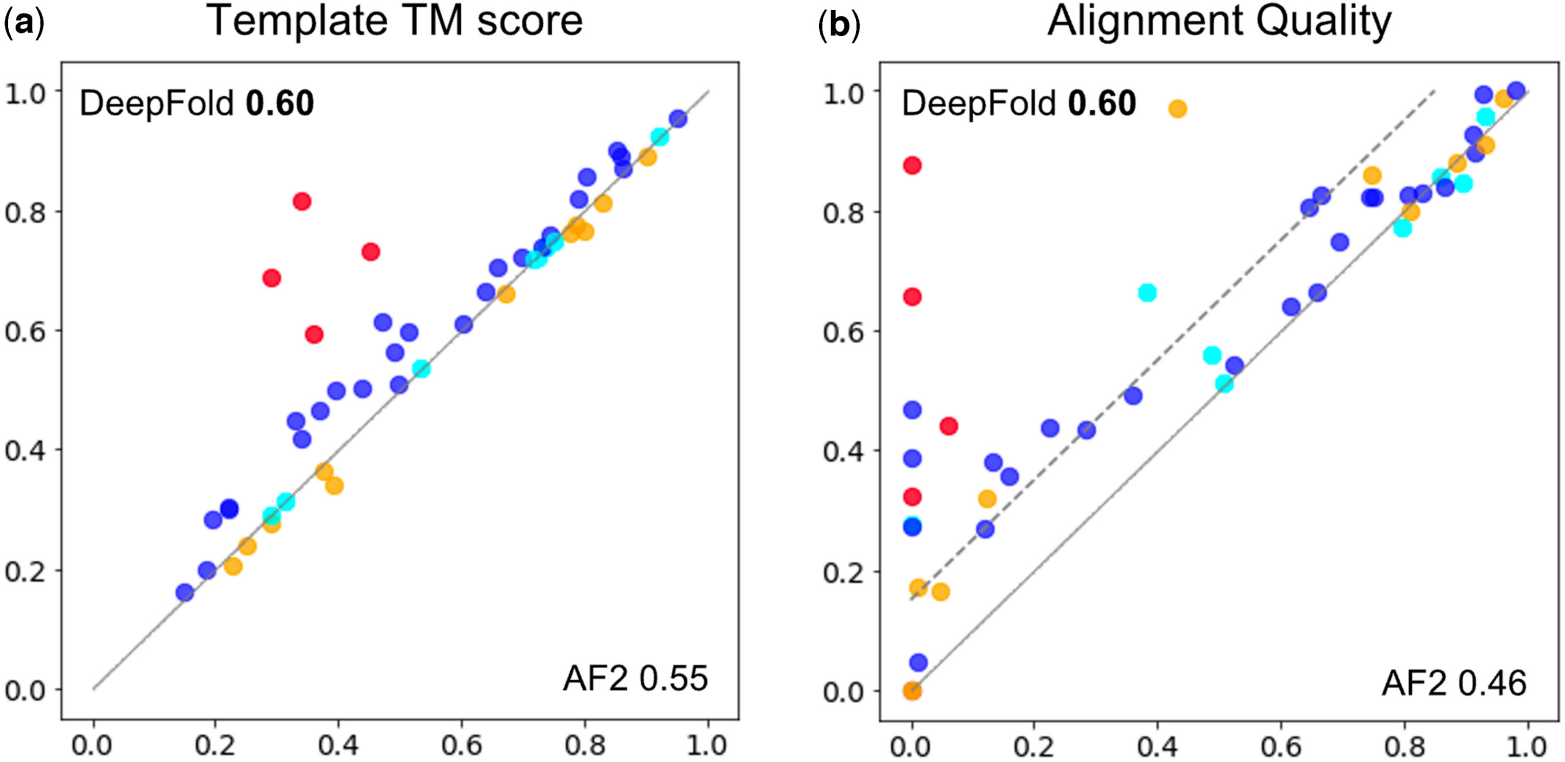
Comparison of the templates of the 55 domains in CASP15. (a) TM-score comparison is shown between the templates found by DeepFold (through CRFalign) versus the ones found by AF2. Each point represents the template with the highest TM-score obtained by the respective method for a given target. Orange colors are the domains with a lower TM-score for CRFalign than the AF2 method, and cyan colors indicate the domains for which the same templates are found by both methods. Red color is the target T1123. (b) A comparison of the alignment qualities of CRFalign and AF2 is shown. All numbers within the figures are average values over 55 domains. The dotted line is indicating a difference > 0.15, and as a result, we had significantly better template information for 18 domains.

**Figure 6.**
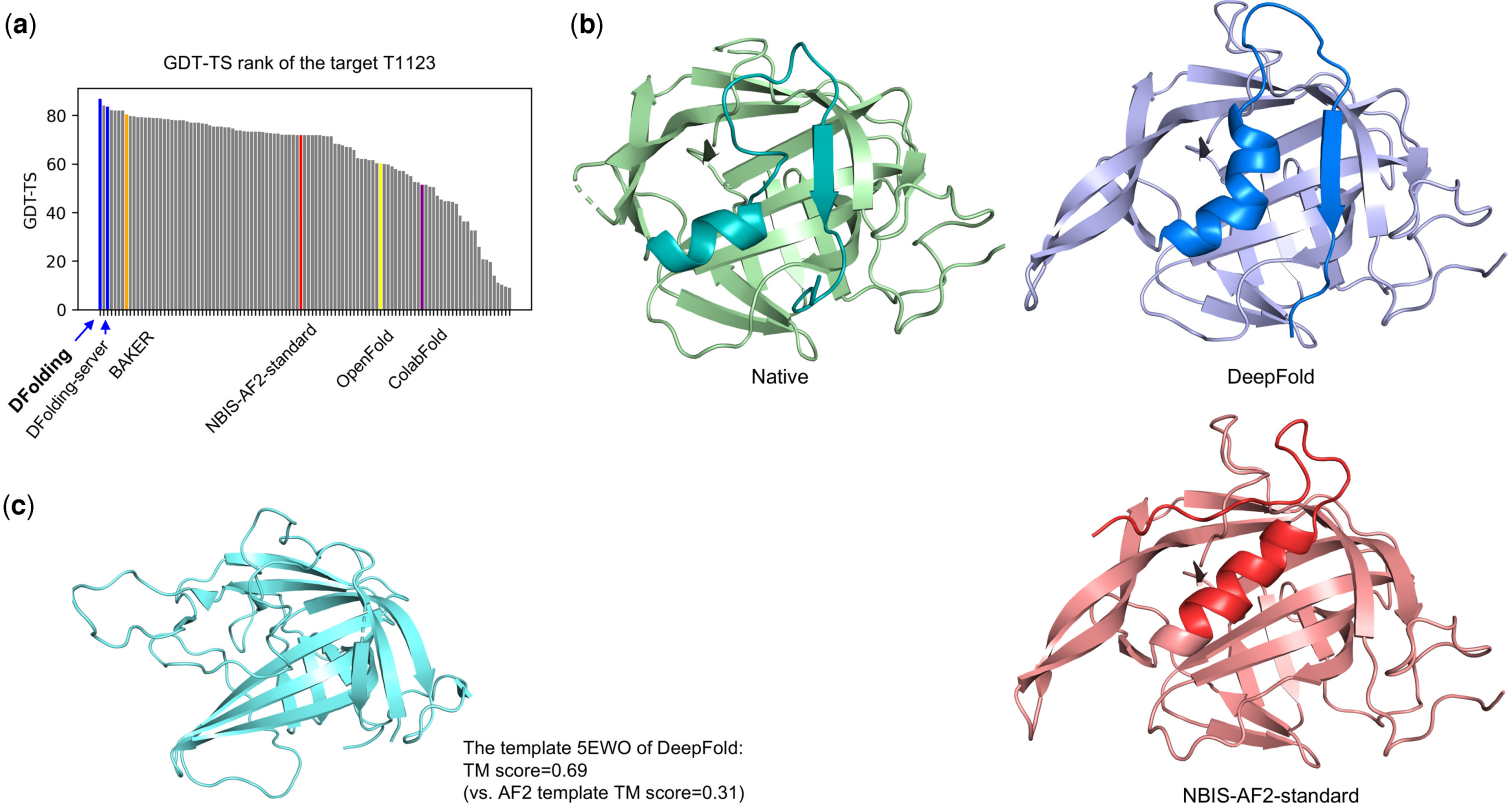
The target T1123 in CASP15 for which DeepFold predictions showed the best GDT-TS scores. (a) DeepFold human and server models ranked at the top three in a row. (b) The predicted 3D structures of T1123 and the native structure (colored as green). Compared to AF2, the DeepFold prediction correctly predicts helix and β-sheets near the C-terminal region, highlighted in blue. (c) The template 5EWO obtained by CRFalign shows a high structural similarity to the native structures (TM = 0.69) compared with the template in AF2 (TM = 0.31).

As mentioned in the Section 2, DFolding used CRFalign for the generation of template features. We can evaluate the quality of template features for 55 domains where the native structures are available. Figure 5 shows the difference between template features of DFolding and those of AF2. In order to show the difference between DFolding and AF2 in terms of the quality of templates, we used the best template among the (up to) four templates for each target. Figure 5a shows the TM-scores of the templates of DFolding in comparison with those of AF2’s templates. Average TM-score of DFolding templates was 0.6042 while that of AF2 was 0.5521. Figure 5b shows a comparison of the quality of CRFalign alignments and those of AF2’s. The alignment quality was measured based on the pairwise distance of residues. First, for the native sequence, collect pairs of residues and measure pairwise distances. The collected pairs are >3 in sequence separation, with mutual distances <8 Å. Second, for the residue pairs at the same location with the native sequence, pairwise distance is calculated for the aligned template sequence. Then, we calculate the ratio of the template residue pairs that have a similar distance to the native residue pairs’ distance. If the difference of a residue pair distance between native and template is less than a threshold, we label that the template residue pair distance is similar to that of native’s. In the case of (b), we set the threshold as 3 Å. We can see that the alignment quality of DFolding using CRFalign shows significant improvement over that of AF2, especially in the cases of hard targets (when the ratio of alignment quality is less than about 0.7). However, because the predicted structure is greatly affected by the quality of the MSA, the superiority of template information does not guarantee the improvement of the final structure. To further verify this, we added the final results of the 18 domains in Fig. 5b where the difference in values is >0.15 in Supplementary Section S7. For example, Target T1123, which belongs to the FM/TBM class, was among the relatively well-predicted cases. As illustrated in Fig. 6a, DFolding ranked first with a GDT-TS score of 86.80, while DFolding-server was placed in the third position with a GDT-TS score of 83.64, outperforming other groups. Figure 6c shows the best template, 5EWO (York *et al.* 2016), which was found using CRFalign. The template exhibited higher structural similarity to the native structure (TM-score = 0.69) than AF2 (TM-score = 0.31). Figure 6b displays the predictions of DFolding and AF2 in cartoon style with the native structure. It can be observed that our models successfully generated β-sheets (shown in blue color) near the C-terminal, while AF2 failed to predict that region.

### 3.1 What went wrong

DFolding failed to generate correct fold for some targets especially for T1125 and T1130. T1125 was a challenging target, likely due to its large size of 1200 residues, which consists of 6 domains (five FM domains and one TBM-hard). Only a few groups achieved good scores (https://predictioncenter.org/casp15/results.cgi?view=tb-sel). For T1125-D1, the average GDT-TS of all groups was 28.44, but that of top three groups had scores around 80. The Neff score of MSA [i.e. represents a measure of the diversity or the effective number of sequences within the alignment, with higher scores indicating a more diverse set of sequences in the MSA (Meier and Söding 2014).] generated by AF2 standard was 1.39 with only five sequences including the target itself, resulting in low plddt scores ranging from 0.39 to 0.44. For the single domain target T1130 with 198 residues (FM domain), DFolding exhibited poor performance with 29.40 of GDT-TS along with most of the groups, while only 3 groups achieved around 95. For this target, the Neff score of AF2 is 0.0 with no additional homologous sequences. And no template structures were found as can be expected for an FM domain. It is highly probable that the three successful groups identified high-quality MSAs through their extended efforts for the target. We also attempted to generate MSAs using other available web servers, but with limited success. As a result, it appears that MSA quality is crucial for the successful prediction of structures using AF2-based methods.

### 3.2 What went right

We found that the improvement of DeepFold’s prediction accuracy can be attributed to the following three elements: modified loss functions, choice of better templates, and CSA re-optimizations. To begin with, the positive effect of modified loss functions is confirmed by ablation studies, as shown in the Supplementary Section S3. To summarize, modified loss functions improved the side-chain accuracy while maintaining the backbone accuracy. The choice of better templates and alignments (partly through CRFalign) improved the backbone accuracy of the specific targets. Finally, CSA re-optimization improved backbone accuracy and molprobity scores through structure optimization.

## 4 Conclusion

In this work, we have presented DeepFold, a pipeline for predicting protein structures. Our primary objective was to improve the accuracy of backbone and side-chain prediction, and we achieved this by making several modifications to the AF2 pipeline. These modifications included optimizing the loss functions with focus on torsion angle losses and additional loss functions introduced to improve side chain accuracy and secondary structure prediction with updated template features. Further re-optimization was performed by CSA using a molecular mechanics energy function which integrates the potential energies obtained from distogram and side-chain prediction. In the blind test at the CASP15 competition, DeepFold improves the details of the protein structure in both backbone and side-chains, ranking fourth in the category of Single Protein and Domain Modeling (109 domains). In addition, DeepFold’s structure prediction showed the best results in terms of backbone accuracy of GDT-TS for the targets in the TBM class (62 among 109 domains), which are more important in practical applications. Our thorough analysis of 55 domains with publicly available ground-truth structures revealed that DeepFold showed the best side-chain accuracy and Molprobity scores in the top-performing groups. It can be seen that the effect of modification and retraining of the DeepFold network and the final re-optimization have made significant differences. In particular, it can be seen that re-optimization further improves the details of protein structure such as backbone, side-chain and Molprobity.

There remain limitations in predicting certain targets, such as very large target like T1125, and T1130 both with low-quality MSAs. This is due to the well-known fact that the performance of AF2-based methods is heavily dependent on the quality of MSAs. It is essential to evaluate the quality of the obtained MSA and enhance the quality of low-quality MSAs by employing techniques such as protein language models (Lin *et al.* 2023) or generative models (Liu *et al.* 2022). Furthermore, in order to predict larger protein structures properly, reliable protein domain parsing must be considered.

In conclusion, our work on DeepFold represents an improvement upon the foundation laid by AF2. Through our modifications to the AF2 pipeline, we have achieved higher accuracy in terms of backbones and side-chains. We believe that our work has advanced the field of protein structure prediction, which we expect to be a valuable tool for the protein modeling community.

## Supplementary Material

btad712_Supplementary_Data

## Data Availability

The data underlying this article is available at https://github.com/newtonjoo/deepfold.
