# Correction to: DeepFold: enhancing protein structure prediction through optimized loss functions, improved template features, and re-optimized energy function

**DOI:** 10.1093/bioinformatics/btad762

**Published:** 2023-12-22

This is a correction to: Jae-Won Lee, Jong-Hyun Won, Seonggwang Jeon, Yujin Choo, Yubin Yeon, Jin-Seon Oh, Minsoo Kim, SeonHwa Kim, InSuk Joung, Cheongjae Jang, Sung Jong Lee, Tae Hyun Kim, Kyong Hwan Jin, Giltae Song, Eun-Sol Kim, Jejoong Yoo, Eunok Paek, Yung-Kyun Noh, Keehyoung Joo, DeepFold: enhancing protein structure prediction through optimized loss functions, improved template features, and re-optimized energy function, *Bioinformatics*, Volume 39, Issue 12, December 2023, btad712, https://doi.org/10.1093/bioinformatics/btad712

In the originally published version of this manuscript, acknowledgment of a funding source is missing. The additional funding number, ‘2020-0-01373,’ has been included in the acknowledgment section. The correct funding acknowledgement is as follows:

This work was supported by Institute of Information & communications Technology Planning & Evaluation (IITP) grant funded by the Korea government (MSIT) [2021-0-02068, 2020-0-01373]; the Basic Science Research Program through the National Research Foundation of Korea (NRF) funded by the Ministry of Science and ICT (MSIT) [NRF-2018R1D1A1B07049312] and the 2023 KT ICT AI2XL Laboratory R&D Fund project funded by KT [KT award B230000177].

This error has been corrected online.

