# Supplementary material for: DeepFold: enhancing protein structure prediction through optimized loss functions, improved template features, and re-optimized energy function
Source: Bioinformatics. 2023 Nov 23;39(12):btad712. doi: 10.1093/bioinformatics/btad712 (PMC10699847; doi:10.1093/bioinformatics/btad712)
Supplement: btad712_Supplementary_Data [file btad712_supplementary_data.pdf]

DEEPFOLD: enhancing protein structure prediction through optimized loss functions, improved template features, and re-optimized energy function

Supplementary information

Jae-Won Lee[1,3†], Jong-Hyun Won[1,3†], Seonggwang Jeon[1,3†], Yujin Choo[2,3†], Yubin Yeon[1,3†], Jin-Seon Oh[2,3†], Minsoo Kim[4†], SeonHwa Kim[5], InSuk Joung[6], Cheongjae Jang[1], Sung Jong Lee[7], Tae Hyun Kim[1], Kyong Hwan Jin[5], Giltae Song[8], Eun-Sol Kim[1], Jejoong Yoo[4], Eunok Paek[1], Yung-Kyun Noh[1,9*], and Keehyoung Joo[3*]

[1]Department of Computer Science, Hanyang University, Seoul, Korea
[2]Department of Artificial intelligence, Hanyang University, Seoul, Korea
[3]Center for Advanced Computation, Korea Institute for Advanced Study, Seoul, 02455, Korea
[4]Department of Physics, Sungkyunkwan University, Korea
[5]School of Electrical Engineering, Korea University, Korea
[6]Standigm Inc., 70 Nonhyeon-ro 85-gil, Gangnam-gu, Seoul, Korea
[7]Basic Science Research Institute, Changwon National University, Korea
[8]School of Computer Science and Engineering, Pusan National University, Korea
[9]School of Computational Sciences, Korea Institute for Advanced Study, Seoul, 02455, Korea

1. Dataset, training, and validation

We used the latest PDB database (Feb. 2022) for training. We clustered the sequences of PDB using CD-HIT with 40% sequence identity, which resulted in 31,911 protein chains. We further filtered 23,366 chains of high-resolution (with < 2.5 Å) for a fine-tuning dataset. The obtained sequences were cropped to 256 and 384 residue sizes as in the AF2 for training. Five DeepFold models were selected from training with various training schedules. For all the trained models, we employed the Uni-fold (a trainable version of AF2) training system, where the trainings were started from the AF2 parameters and then further optimized in the style of transfer learning. Table S1 shows the training detail of a representative model for which a validation result is shown below. Details of the five DeepFold models are described in Supplementary Section 6.
For validation, we tested the trained model on 102 targets of CASP13/14. In order to measure the performance on the side chain torsion angles precisely, we defined new accuracy measures χ^1_{acc} for χ^1 and χ^2_{acc} for χ^2. χ^1_{acc} is defined as the proportion of predicted χ^1 angles that have a difference of 10 degrees or less when compared to the ground truth χ^1 angles. For χ^2 accuracy, χ^2_{acc} is defined as the proportion of residues with angles for both χ^1 and χ^2 have a difference of 10 degrees or less with compared to the ground truth χ^1 and χ^2 respectively. Figure S1 shows a comparison of DeepFold predictions with AF2 in backbone, side-chain and secondary structure accuracies. Blue color indicates that DeepFold outperformed AF2, whereas yellow denotes the opposite cases. From Figure S1 (a), we can see that the DeepFold predicts quite different protein structures from those of AF2 for about 13 targets. On the other hand, the average TMscore of DeepFold predictions is 0.8648 while that of AF2 is 0.8592 indicating modest improvement of 0.56% in favor of DeepFold.

Table S1: Training settings and the weights of the loss terms for the training of a DeepFold model

**Training settings**	
Training dataset	384 crop (23,366 chains)
Iteration	3,000
Optimizer	Adam (*Kingma and Ba*, 2014)
Number of Parameters	≈ 89,250,000
**Losses**	**weights**
$\mathcal{L}_{\text{angle}}$	1.0
$\mathcal{L}_{\text{WFAPE}}$	0.5
$\mathcal{L}_{\text{secondary}}$	0.05
$\mathcal{L}_{\text{sc}}$	0.1
$\mathcal{L}_{\text{others}}$	default as standard AF2 (*Jumper, et al.*, 2021)

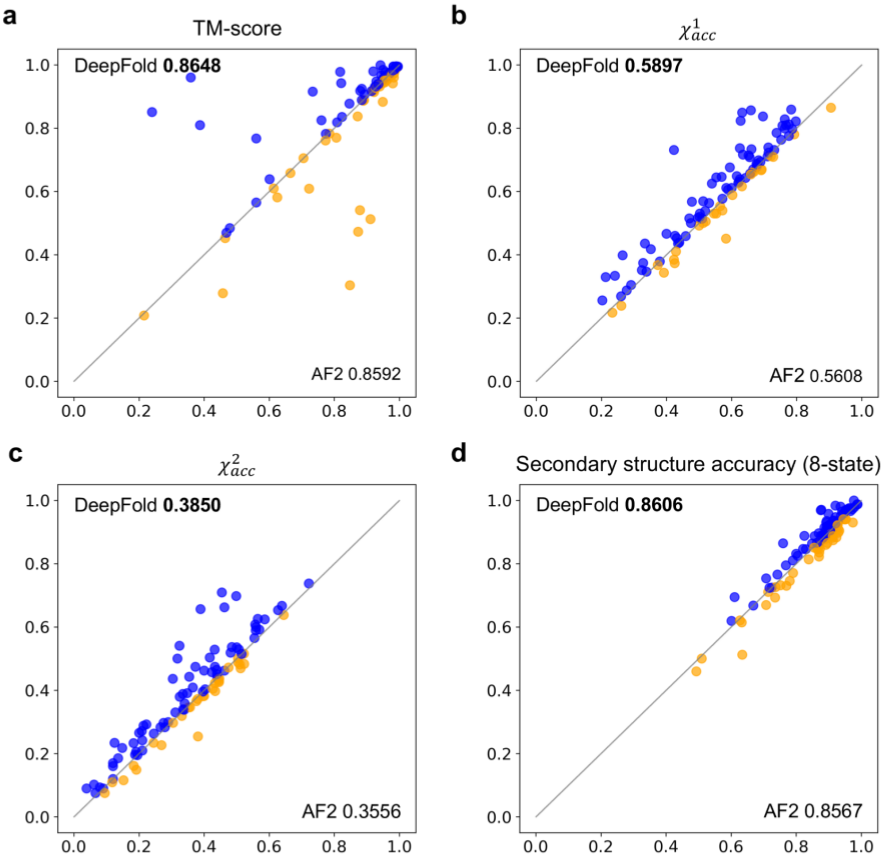

Figure S1: Comparison of DeepFold with AF2 for 102 targets of CASP13/14 in terms of (a) TM-scores, (b) & (c) side chain accuracies of χ^1 and χ^2, and (d) secondary structure accuracies in 8-state (calculated by DSSP). All numbers within the figures are average values over 102 targets.

Figure S1 (b) and (c) show χ^1_{acc} and χ^2_{acc} of DeepFold and AF2, respectively. As can be seen from the plots, the average side chain accuracies of both χ^1 and χ^2 are higher in DeepFold, showing that mean scores are improved about 3% point over AF2 in both accuracy measures. More importantly, the majority of targets are improved consistently in side-chain angles. This implies that the modified loss functions were effective in improving the side chain angle predictions. Figure S1 (d) compares the accuracies of secondary structure prediction on CASP 13/14 targets by DeepFold vs. AF2, which shows that the average secondary structure accuracy of DeepFold (with mean accuracy of 0.8606) was slightly higher than that of AF2 (mean accuracy of 0.8567). Considering that the typical classification accuracy of eight-state secondary structure in the literature ranges from 0.70 to 0.80 (*Spencer*, *et al.*, 2015; Wang, et al., 2016; *Zhang*, *et al.*, 2018), the accuracy of 0.8606 is significantly higher than the typical average accuracy. In addition, we found that (data not shown here) both DeepFold and AF2 showed better or comparable accuracies in all eight states, with both models performing well in predicting 'H' (alpha helix) and 'E' (strand), while they demonstrated low accuracies in predicting 'S' (Bend). Figure S2 (a) compares the predicted average side-chain confidence score for each target $\hat{s}$ and its ground truth value s. As can be seen from the linear fit, a reasonable correlation between the prediction $\hat{s}$ and the true confidence score s was achieved with the correlation coefficient of r = 0.7. Considering that the side chain confidence s reflects the side chain angle differences by definition as shown in Eq. (9), the high correlation implies that the predicted $\hat{s}$ can be used as a good measure for confidence level for side chain accuracy. Figure S2 (b) & (c) are showing a comparison of the pLDDT and LDDT score of all the targets with DeepFold and AF2 respectively. We can see that DeepFold shows a slightly better correlation coefficient of 0.80 over 0.78 of AF2.

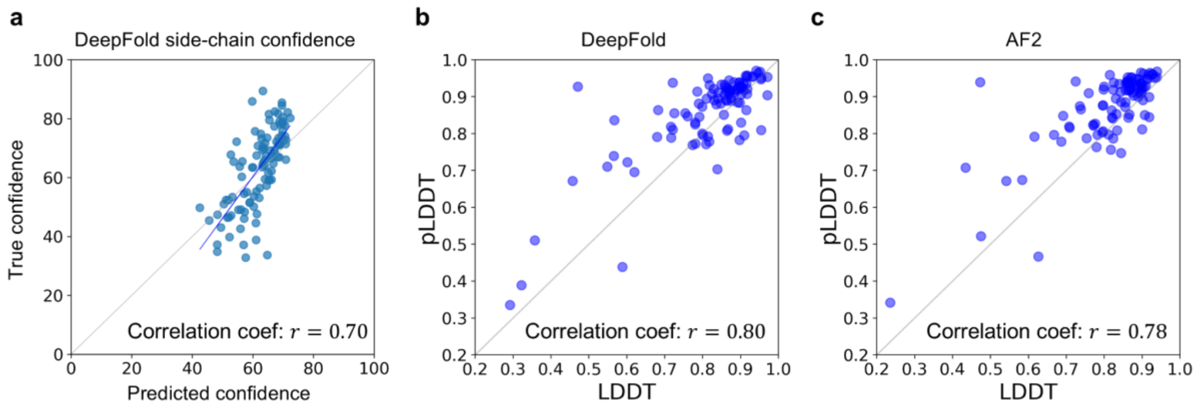

Figure S2: Validation of the trained model on 102 targets of CASP13/14. (a) compares the true average side chain confidences and its prediction $\hat{s}$ for each target, and (b) & (c) compares (normalized) pLDDT vs. LDDT for all the targets by DeepFold and AF2 respectively.

2. Re-optimization by conformational space annealing

Once 3D structures are inferred from the DeepFold networks, we perform global optimization using conformational space annealing (CSA) with the full atom force field, distance restraints, and side chain torsion restraints generated by the networks. The energy function used for CSA is implemented in the PyCSA (*Joung, et al.*, 2018) global optimization library and OpenMM (*Eastman, et al.*, 2017), a molecular dynamics simulation toolkit. The energy function is defined as follows:

$$E = E_{mm} + 0.5 \cdot E_{disto} + E_{side} + 0.2 \cdot E_{stap} \quad \text{(S1)}$$

where E_{mm} is molecular mechanics force field composed of AMBER14SB (*Maier, et al.*, 2015) energy and Generalized Born implicit solvation energy (*Onufriev, et al.*, 2004). The distogram potential energy Edisto is defined by $E_{disto} = -\Sigma_{<ij>} ln\,(p_{ij}/p_{ij}^0)$, where p_{ij} indicates the distogram probability between the residues i and j for $d_{ij} < 17.94$ Å with constant extrapolation for farther distances (*Senior, et al.*, 2020), where p_{ij}^0 is the distogram probability at the cut-off distance. Each term of E_{disto} is an interpolated fit to a cubic spline. Since distogram predictions for larger distances are not expected to be accurate enough, we limited the summation on $< ij >$ over all pairs for which the distance of maximum probability is less than 16.06 Å. E_{side} for the set of all the side-chain torsion angles $\{\phi\}$ is the flat-bottomed Lorentzian-type potential energy (*Joo, et al.*, 2018) defined as follows:

$$E_{side} = \Sigma_k \begin{cases} 0 & \hat{\phi} - \phi_t < \phi < \hat{\phi} + \phi_t \\ w_k \cdot \Delta\phi^2/(\Delta\phi^2 + \sigma^2) & otherwise, \end{cases} \tag{S2}$$

where $\hat{\phi}$ is *k*-th predicted torsion angle obtained from DeepFold, ϕ_t is a tolerance angle (5° in this work), and $\Delta\phi = \left(\left|\phi - \left(\hat{\phi} - \phi_t\right)\right|, \left|\phi - \left(\hat{\phi} + \phi_t\right)\right|\right)$, which results in a flat-bottom of width 10° with Lorentzian-width of σ = 15°. For the weights w_k, we choose {3.0,2.5,2.0,1.5} for the four types ($\chi_1, \cdots, \chi_4$) of sidechain angles respectively. For multiple predicted models, we can generalize the above formula by clustering the predicted angles with a threshold of 30°, and take the new $\hat{\phi}$ as the average of all the predicted angles of each cluster. Also a new σ is taken as $\sigma = max\,(15, max\_dif + 10)$, where max_dif is the maximum angle difference of the cluster. E_{stap} is a statistical potential for pairs of torsion angles including backbone and side-chains (*Yang, et al.*, 2012). The CSA method aims to find the lowest energy structure by exploring its conformational space. In its early stages, CSA searches the entire conformational space, gradually narrowing the search to smaller regions with lower energy. As a result, the CSA method provides the optimized structures that satisfies the distogram and side-chain restraints obtained from DeepFold, while also balancing the molecular mechanics force field.

3. Ablation study for DeepFold

We retrained the DeepFold model to investigate the effect of each modified loss component. The training database was built using the PDB structures deposited before 28 August 2019, the same as that of AlphaFold2 (AF2) (Jumper *et al.*, 2021). It was also filtered with 40% sequence similarity in the same manner as AF2. We also applied the same sequence similarity filter to remove PDB chains similar to CASP13/14 targets. The dataset for the ablation study contains 25,777 chains. In the case of the template database provided by AF2, we filtered template structures based on the start dates of CASP13/14, respectively.

We trained 3 ablation models as follows:
W-FAPE: We keep the weighted W-FAPE loss term (eq. 6) in the loss configuration.
SC-torsion: We keep our proposed side-chain losses which are sequentially dependent. We note that the original AF2 side-chain loss was kept as default.
Full model: Full DeepFold model as described in the paper.

We excluded secondary structure loss for the ablation study due to the very small weight. It was designed only for predicting secondary structure as a reference. At the start of training, our models are initialized from AlphaFold2 model 1 parameters, which are then further trained for 30 epochs for each model configuration. We set the gradient of the evoformer model to zero and only trained the structure module, as our configuration for the DeepFold model. Training each model required approximately 3 days using a batch size of 64 on eight A100 GPUs. We employed the Adam optimizer with a learning rate set to 1e-3.

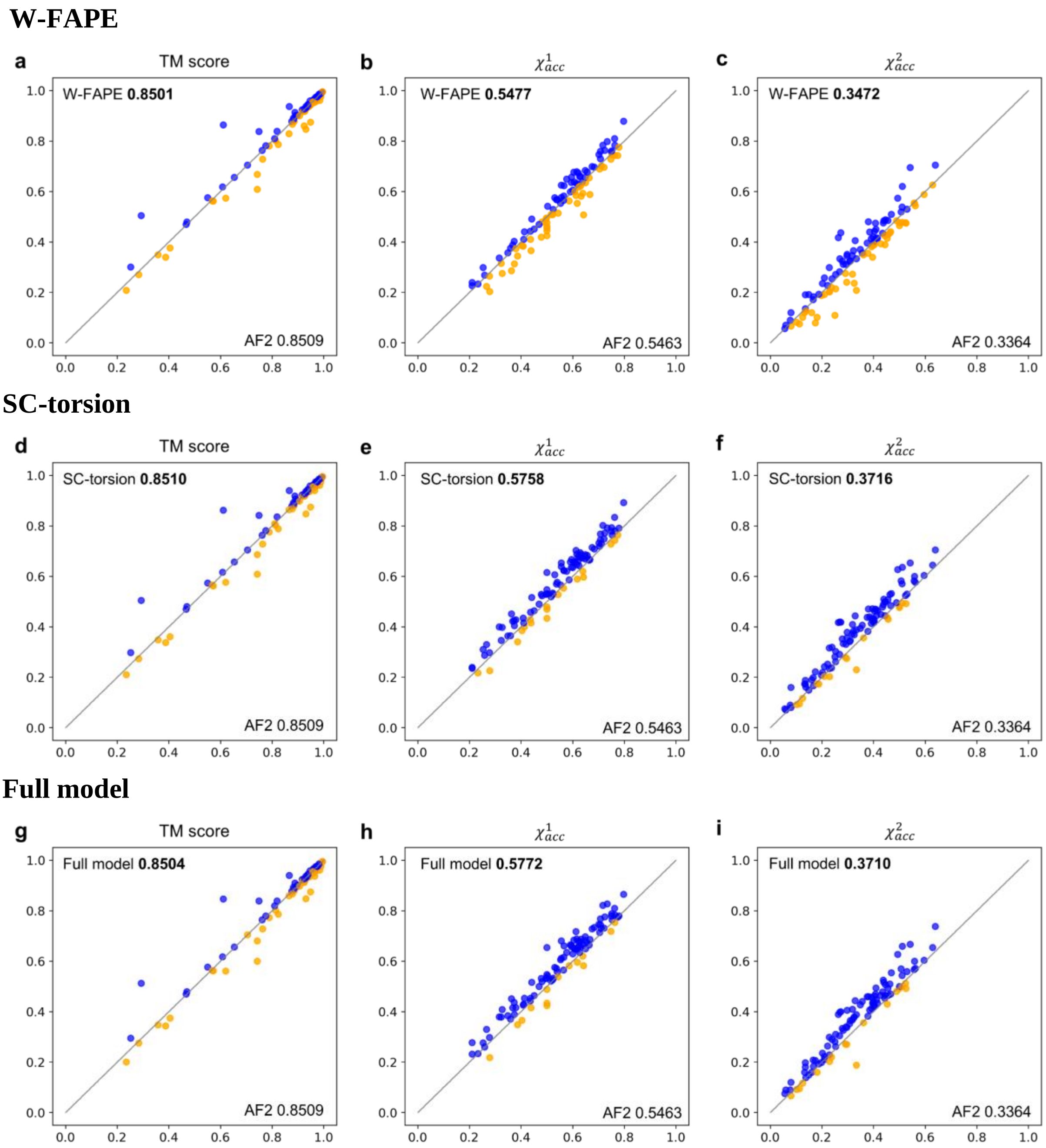

Figure S3: Comparison of ablation models with the AlphaFold2 model. We validate the trained models with 102 CASP 13/14 targets. The first row shows the results for W-FAPE loss, while the second and third row represent the results for SC-torsion loss and those of the full model respectively.

Figure S3 presents the outcomes of the ablation study for the three aforementioned models. Even with just the W-FAPE or SC-torsion loss, there are improvements in side-chain accuracy. When these two losses are combined in the full model, the side-chain accuracy is further improved. Detailed numerical results are provided in Table S2. In conclusion, the DeepFold architecture elevates the side-chain accuracy, while the backbone accuracy remains stable. Note that the Molprobity score gets worse from ~1.06 to ~2.3 (small is better). However, it was improved through later CSA re-optimization. (See the Molprobity comparison between DFolding-server and DFolding in the Figure 3 of the main manuscript.)

Table S2: Validation of CASP13/14 targets with various metrics. We observe that modified side chain loss is effective in increasing chi1, chi2 accuracies. Also, we find that combining the two losses gives better results in side-chain accuracies.

Model	TM-score	LDDT	Chi1	Chi2	MPScore
AF2	0.8509	**0.8183**	0.5463	0.3364	1.0645
W-FAPE	0.8501	0.8139	0.5477	0.3427	2.3001
SC-torsion	**0.8510**	0.8138	0.5758	**0.3716**	2.3886
Full model	0.8504	0.8142	**0.5772**	0.3710	2.3287

4. Impact of templates for the CASP13/14 targets

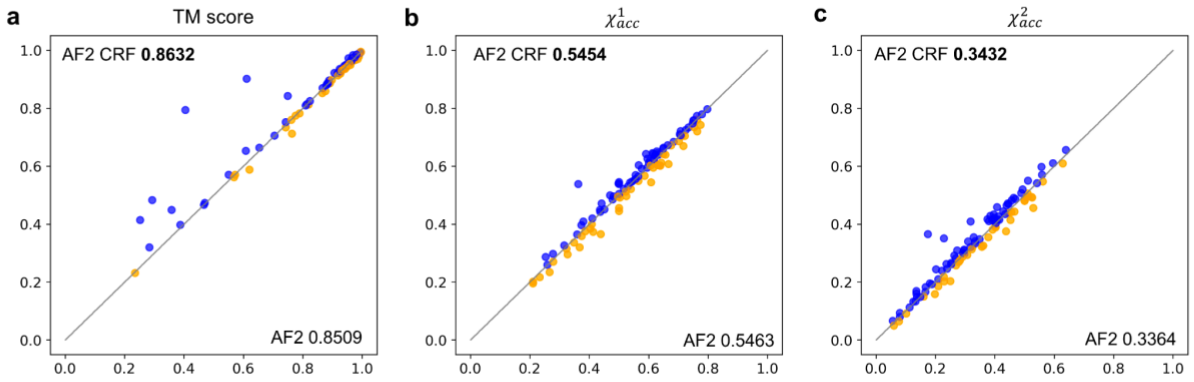

Figure S4: TM-score Comparison between AF2-baseline templates vs. CRFalign templates and their alignments.

Figure S4 (a) illustrates a TM-score comparison between the case of the original AF2 templates and that of new templates (and their alignments), which were obtained by using the CRFalign method (*Lee, et al.*, 2022). Structures were generated using the AF2 pipeline. For the 102 CASP13/14 targets, there is an average TM-score improvement of about 0.01. Meanwhile, certain targets display substantial improvement in head-to-head comparison. Specifically, for T1064, the TM-score improvement is 0.39. In Figure S4 (b, c), it shows that the side-chain prediction remains largely unchanged despite the template change.

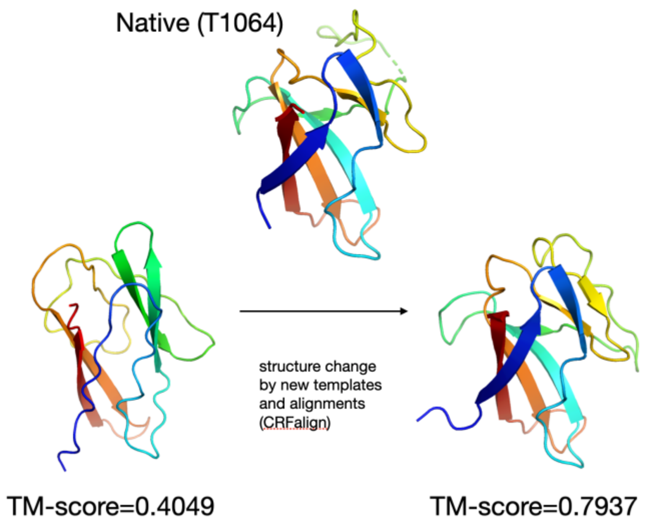

Figure S5: Structure change by new templates and alignment for T1064.

As depicted in Figure S5, the backbone improvement for the predicted structure (by DeepFold through CRFalign) on target T1064 is shown with a TM-score difference of about 0.39 over that by AF2 (from 0.4049 to 0.7937). For this target, the Neff score of the MSA was relatively low, around 1.85, and the average TM-score difference among the top 4 templates was 0.11 (0.31 vs. 0.42) which is a considerable difference but not a huge one. Still, there was a considerable TM-score difference of approximately 0.39 in the final result. When comparing protein tertiary structures, notable differences can be observed in the beta-sheet arrangement of the intermediate regions, ranging from cyan to yellow colors, when compared with the native structure. The impact of templates on protein structure prediction accuracy was studied in a recent work (*Wu, et al.*, 2023) where they concluded that templates are especially beneficial for targets with similar templates.

5. Comparison between CSA re-optimization and AF2 recycle

Table S3: Note that AF_# refers to the AF2 with # times iteration.

Methods	TM-score	GDT-TS	LDDT	$S_{acc}(\chi_1)$	$S_{acc}(\chi_2)$	Molprobity
**CSA**	**0.8878±0.1453**	**86.61±12.80**	**0.8422±0.0590**	**0.6132±0.1396**	**0.4042±0.1145**	**0.6147±0.1165**
AF_4*	0.8837±0.1341	85.58±12.26	0.8329±0.0773	0.5823±0.1366	0.3809±0.1237	1.0999±0.2986
AF_8	0.8841±0.1370	85.78±11.95	0.8362±0.0668	0.5825±0.1391	0.3739±0.1304	1.0950±0.3367
AF_12	0.8825±0.1369	85.67±11.98	0.8331±0.0764	0.5786±0.1404	0.3784±0.1318	1.0962±0.2914
AF_16	0.8812±0.1373	85.37±12.17	0.8317±0.0771	0.5811±0.1436	0.3713±0.1340	1.0830±0.3020
AF_20	0.8833±0.1375	85.85±12.15	0.8344±0.0748	0.5764±0.1360	0.3769±0.1315	1.1249±0.3131

Using the domains for which PDBs are publicly available, we performed comparisons between the results of CSA reoptimization and those of AF2s with several different iterations. The result is shown in the following table which indicates that the CSA method produces better average scores on side-chain torsion angles and molprobity. This demonstrates that CSA reoptimization is an appropriate tool for advancing the details of protein structures, as we suggested in the main paper. Apparently, increasing the number of recycling iterations does not lead to statistically significant changes in AF2 results beyond four times as AF2 paper reports (*Jumper et al.*, 2021). However, another research group as ColabFold (*Mirdita, et al.*, 2022) suggests that increasing the number of recycles can be beneficial for larger proteins or complex structures.

6. Training details

We trained each DeepFold model with 8 A100 GPUs, where the batch size was 64. Because the batch size is larger than the number of GPUs, we used gradient accumulation for training. The training time for each model is about 3-4 days. We employed a fine-tune strategy for model 0~3, freezing the evoformer module with the gradients affecting only the structure module. For our model training, the differences in hyperparameters between DeepFold models are outlined in TableS3. We trained the initial model with our recent PDB dataset. This initial training did not include our proposed modifications to loss functions. Then we further trained our model with bigger crop sizes together with our proposed loss functions. We used the Adam optimizer with exponential decay for which decay_rate=0.95 and decay_steps=500.

Table S3: Training Details for 5 DeepFold models.

	Initial	Fine-tuning				
models	0	1	2	3	4	5
Parameter Initialization	AlphaFold Model 1	DeepFold Model 0	DeepFold Model 0	DeepFold Model 0	AlphaFold Model 3	AlphaFold Model 4
crop size	256	384	384	384	384	384
learning rate	1e-3	1e-4	1e-4	1e-4	1e-4	5e-5
Evoformer train	False	False	False	False	True	True
Template Feature	True	True	True	True	True	False
Training Time	~ 4 days	~ 3 days	~ 3 days	~ 3 days	~ 3 days	~ 3 days
L_{angle} weight	0.5	1.0	1.0	1.0	1.0	1.5
L_{angle} dependent	False	True	True	True	True	True
L_{WFAPE} weight	0	0.5	1.0	1.0	1.5	2.0
$L_{Secondary}$	0.0	0.05	0.01	0.0	0.0	0.0

7. Further investigation of template effects in CASP15

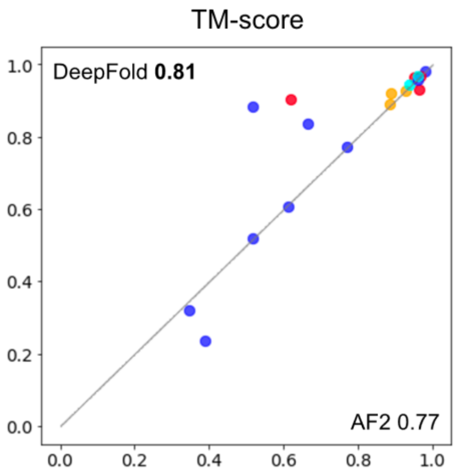

Figure S6: Comparison of TM-scores between AF2 and DeepFold for the 18 selected domains. These domains are depicted above the dotted line (indicating a difference > 0.15) in Figure 7(b) of the main manuscript. (T1176-D9, T1137s4-D1, T1123-D1, T1159-D1, T1137s4-D3, T1187-D1, T1104-D1, T1137s3-D2, T1120-D1, T1145-D1, T1114s1-D2, T1121-D1, T1119-D1, T1121-D2, T1152-D1, T1137s6-D2, T1106s1-D1, T1122-D1)

Figure S6 illustrates a comparison of TM-scores between AF2 and DeepFold for 18 domains. These domains are depicted above the dotted line (indicating a difference > 0.15) in Figure 7(b) of the main manuscript. On average, TM-score improved from 0.80 in AF2 to 0.83 in DeepFold. In particular, three of these domains (T1123-D1, T1137s6-D2, and T1106s1-D1) show significant TM-score improvement, demonstrating the

effectiveness of our methodology. For example, domain T1123-D1, shown in red, showed significant improvement despite having a Neff value of 1.9, indicating relatively low MSA quality. In such cases, our data suggest that DeepFold's updated template information can help improve prediction results. However, Figure 7(a) in the main manuscript shows that there is no improvement for the other three domains marked in red. These three domains all had Neff values greater than 6.0, making them high-quality targets for MSA. It is worth mentioning that when the quality of MSA information is high, the impact of templates on the prediction structure may not be significant, as highlighted in the AF2 paper (Jumper *et al.*, 2021).

References

1. Eastman, P., *et al.* OpenMM 7: Rapid development of high performance algorithms for molecular dynamics. *PLoS computational biology* 2017;**13**(7):e1005659.
2. Joo, K., *et al.* Data-assisted protein structure modeling by global optimization in CASP12. *Proteins: Structure, Function, and Bioinformatics* 2018;**86**:240-246.
3. Joung, I., *et al.* Conformational Space Annealing explained: A general optimization algorithm, with diverse applications. *Computer Physics Communications* 2018;**223**:28-33.
4. Jumper, J., *et al.* Highly accurate protein structure prediction with AlphaFold. *Nature* 2021;**596**(7873):583-589.
5. Kingma, D.P. and Ba, J. Adam: A method for stochastic optimization. *arXiv preprint arXiv:1412.6980* 2014.
6. Lee, S.J., *et al.* CRFalign: A Sequence-structure alignment of proteins based on a combination of HMM-HMM comparison and conditional random fields. *Molecules* 2022;**27**(12):3711.
7. Maier, J.A., *et al.* ff14SB: improving the accuracy of protein side chain and backbone parameters from ff99SB. *Journal of chemical theory and computation* 2015;**11**(8):3696-3713.
8. Mirdita, M., *et al.* ColabFold: making protein folding accessible to all. *Nature methods* 2022;**19**(6):679-682.
9. Onufriev, A., Bashford, D. and Case, D.A. Exploring protein native states and large-scale conformational changes with a modified generalized born model. *Proteins: Structure, Function, and Bioinformatics* 2004;**55**(2):383-394.
10. Senior, A.W., *et al.* Improved protein structure prediction using potentials from deep learning. *Nature* 2020;**577**:706-710.
11. Spencer, M., Eickholt, J. and Cheng, J. A Deep Learning Network Approach to ab initio Protein Secondary Structure Prediction. *IEEE/ACM Transactions on Computational Biology and Bioinformatics* 2015;**12**(1):103-112.
12. Wang, S., *et al.* Protein secondary structure prediction using deep convolutional neural fields. *Scientific reports* 2016;**6**(1):1-11.
13. Wu, F., *et al.* Improving protein structure prediction using templates and sequence embedding. *Bioinformatics* 2023;**39**(1).
14. Yang, J.S., *et al.* STAP Refinement of the NMR database: a database of 2405 refined solution NMR structures. *Nucleic acids research* 2012;**40**(D1):D525-D530.
15. Zhang, B., Li, J. and Lü, Q. Prediction of 8-state protein secondary structures by a novel deep learning architecture. *BMC bioinformatics* 2018;**19**(1):1-13.
